# Oral expressions and functional analyses of the extracellular calcium-sensing receptor (CaSR) in chicken

**DOI:** 10.1038/s41598-022-22512-6

**Published:** 2022-10-22

**Authors:** Hikaru Omori, Yuko Kawabata, Yuta Yoshida, Yutaro Nagamoto, Fuminori Kawabata, Shotaro Nishimura, Shoji Tabata

**Affiliations:** 1 Laboratory of Functional Anatomy, Faculty of Agriculture, Kyushu University, Fukuoka, Japan; 2 Department of Food and Life Sciences, Ibaraki University, Ami, Japan; 3 Physiology of Domestic Animals, Faculty of Agriculture and Life Science, Hirosaki University, 3 Bunkyo-Cho, Hirosaki, Aomori, 036-8561 Japan; 4 Present Address: Department of Cell Biology, Aging Science, and Pharmacology, Division of Oral Biological Sciences, Faculty of Dental Science, Kyushu University, Fukuoka, Japan

**Keywords:** Taste receptors, Cellular imaging, Animal physiology

## Abstract

In vertebrates, the extracellular calcium-sensing receptor (CaSR) plays a key role in calcium homeostasis by sensing slight changes in extracellular Ca^2+^. CaSR is also expressed in mammals including rodent taste cells and is involved in sensing *kokumi*, a rich, savory quality that enhances the intensities of salty, sweet, and umami tastes. In this study, we focused on chicken CaSR (cCaSR) since calcium is an essential nutrient that is necessary for making eggshell and for the extremely rapid initial growth of bones. First we confirmed that cCaSR is expressed in taste cells. Next we cloned the c*CaSR* gene from kidney and transiently transfected human embryonic kidney 293 T (HEK293T) cells with the recombinant c*CaSR*, or empty vector and looked for the agonists and allosteric modulators (including *kokumi* substances) of cCaSR by Ca^2+^ imaging. We found that cCaSR was activated by extracellular Ca^2+^ and Mg^2+^ in a dose dependent manner. Several L-amino acids and *kokumi* substances such as glutathione enhanced the response of cCaSR. In addition, NPS2143 as a negative allosteric modulator of human CaSR negatively modulated the response of cCaSR. These results suggest that cCaSR can sense extracellular Ca^2+^ and Mg^2+^ as well as positive and negative allosteric modulators. Taken together, the results imply that CaSR might be a multifunctional receptor for calcium, amino acids, and *kokumi* substances in chicken. The present finding that functional CaSR is expressed in the chicken oral tissues will allow us to further elucidate the physiological role of CaSR in the chickens' taste sense, and to create new feeds that will contribute to the poultry industry.

## Introduction

Calcium is a very important nutrient for chickens. Low levels of calcium in the diet induce decreases in egg production, eggshell strength, hatchability, and body weight gain^1^. It is known that chickens show a high affinity to calcium^2,3^. However, it is unknown whether chickens use taste to discriminate feed containing calcium.

The extracellular calcium sensing receptor (CaSR), a G-protein–coupled receptor belonging to Family C, is abundantly expressed in the parathyroid glands, kidney, gut, and bone and plays a key role in regulating the release of parathyroid hormone in response to circulating blood calcium concentrations in mammals^4^. Extracellular Ca^2+^ is the principal agonist of CaSR^5^, and other orthosteric agonists include various divalent and trivalent cations, including Mg^2+^, Al^3+^, Sr^2+^, Mn^2+^, Ni^2+^, Gd^3+^, and Ba^2+^, as well as polyamines (spermine, spermidine, and putrescine), aminoglycoside antibiotics (neomycin, gentamicin, kanamycin, tobramycin), and some peptides (amyloid β-peptides, poly-lysine, and poly L-Arg)^6,7^. Furthermore, functions of CaSR can be regulated by many allosteric modulators such as aromatic L-amino acids (L-Phe, L-Trp, L-Tyr, and L-His), calcimimetics (Cinacalcet, NPS R-568, NPS R-467, and Calindol), and calcilytics (NPS2143 and Calhex 231)^6,7^.

Tordoff et al. revealed that T1R3 is a calcium-magnesium taste receptor in mice^8^, and it was speculated that *CaSR* is also a calcium-magnesium taste receptor candidate gene^9^. Indeed, CaSR is expressed in taste cells in rodents^10,11^. Moreover, it has been reported that CaSR functions as the receptor of a specific flavor quality, *kokumi*^9,10,12^. Although *kokumi* substances have only a slight flavor in water, they were found to markedly increase thickness, continuity, and mouthfulness of taste when added to an umami solution or other food^13^. These *kokumi* substances, such as glutathione (γ-L-glutamyl-L-cysteinylglycine, GSH), are perceived through CaSR in mice and humans^10,12^.

Elucidation of the functions of chicken CaSR (cCaSR) in oral tissues may reveal fundamental knowledge with which to improve chicken feeds and enhance the efficiency of egg and meat production. Although it has been reported that chickens have umami, bitter, and fat taste receptors^14–23^, it is unknown whether chicken oral tissues have sensors for calcium and *kokumi* flavors. Thus, the purpose of this study was to elucidate the sensing mechanisms of calcium taste and *kokumi* quality in chickens. We analyzed CaSR expression in chicken oral tissues and investigated whether cCaSR is activated by Ca^2+^, Mg^2+^, L-amino acids, and *kokumi* substances by using Ca^2+^ imaging assay by using cCaSR-expressing cells.

## Results

### Reverse transcription qPCR

**Figure 1:**
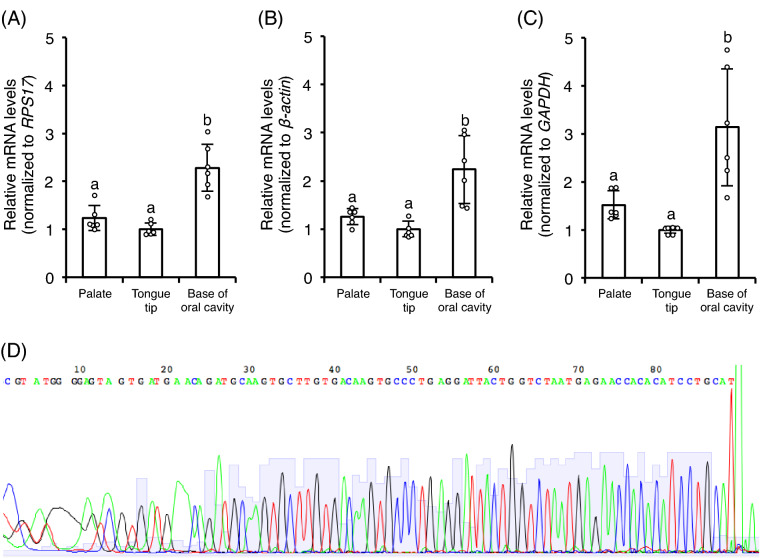
The relative *mRNA* levels of *CaSR* were significantly higher in the base of oral cavity than in the palate and the tongue tip in chickens. Values are the mean relative *mRNA* levels (normalized to (**A**) *RPS17*, (**B**) *β-actin*, and (**C**) *GAPDH*) ± SD (n = 6). Bars without a common letter differ significantly, *P* < 0.01 by one-way factorial ANOVA followed by post-hoc Tukey HSD test. (**D**) Representative sequence analysis data of the RT-*q*PCR product of the base of oral cavity by using CaSR primer pair.

A previous study demonstrated that the taste buds of chickens were located mainly in the palate and the base of oral cavity and that very few were located in the tongue tip, unlike the case in mammals^24^. The present study showed that *CaSR mRNA* expression levels were significantly higher in the base of oral cavity than in the palate and the tongue tip (*P* < 0.01) (Figs 1A–C). Although the expression level in the palate was slightly higher than in the tongue tip, there was no significant difference. These expressions were confirmed by using three different reference genes. We also confirmed the DNA sequences of reverse transcription (RT)-*q*PCR products of base of oral cavity by using CaSR primer pair were identical with c*CaSR* sequence (Fig. 1D).

### Cloning of chicken CaSR from kidney

We cloned the c*CaSR* gene from kidney. After amplification in *E. coli*, we confirmed that the cDNA sequence of the cloned c*CaSR* was nearly identical to that in the nucleotide sequence of the US National Center for Biotechnology Information (NCBI) database (*Gallus Gallus* CaSR, XM_416491.7), with a difference of only two bases (C993T and A1020G; the left sequence of the number is XM_416491.7 and the right one is that of the Rhode Island Red (RIR) strain). The translated product of the cloned c*CaSR* gene matched that of the NCBI database (XP_416491.6).

### Expression of CaSR in taste cells of chickens

**Figure 2:**
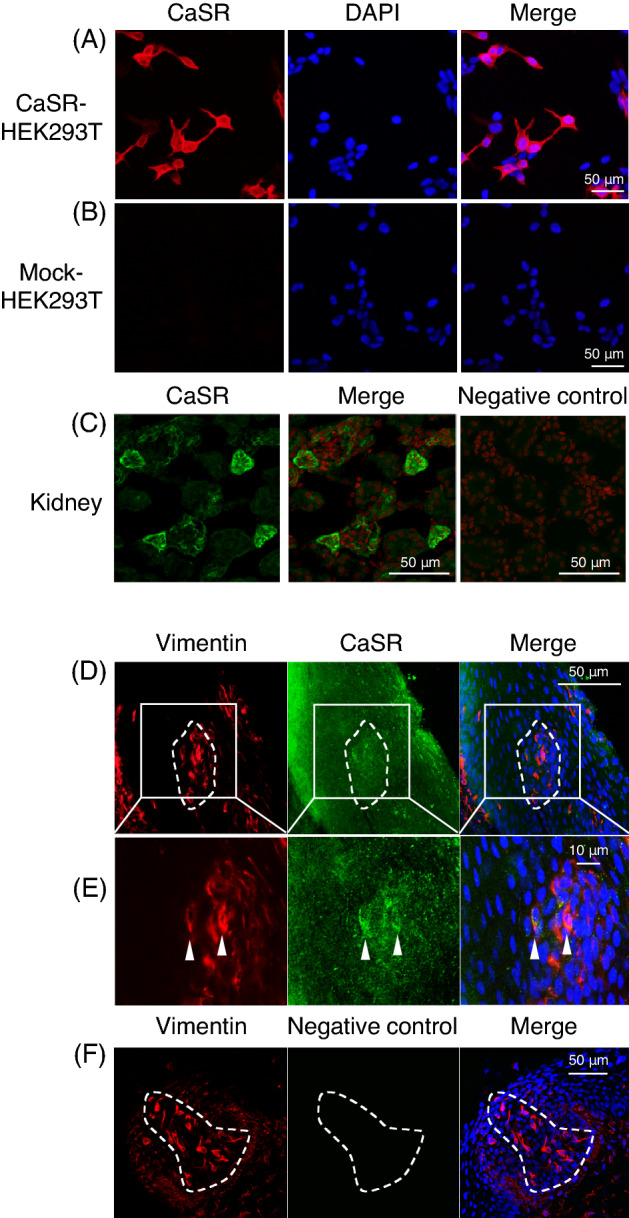
(**A**) HEK293T cells transiently transfected with c*CaSR*/pcDNA5/FRT were immunostained by the cCaSR antiserum generated herein (red). Nuclei were stained with DAPI (blue). (**B**) HEK293T cells transiently transfected with empty vector pcDNA5/FRT (mock) were not labeled by the antiserum. Nuclei were stained with DAPI (blue). (**C**) Expression of CaSR (green) in the kidney in 9-day-old chicks. Ten similar images of kidney were obtained from two chicks. Nuclei were stained with DAPI (red). (**D, E**) Expression of vimentin (red) and CaSR (green) in the taste buds of the base of oral cavity in 7-day-old chicks. Four similar images of the base of oral cavity were obtained from two chicks. One representative image is shown. White dots outline the taste buds labeled by vimentin, the molecular marker for the taste buds of chickens^25^. Nuclei were stained with DAPI (blue). (**E**) shows a magnified image of (**D**). Arrowheads indicate taste cells immunostained by both vimentin and CaSR. (**F**) In the negative control, the antiserum for CaSR was omitted. Nuclei were stained with DAPI (blue).

We investigated the specificity of the anti-chicken CaSR antiserum newly generated in this study. First we observed that human embryonic kidney 293 T (HEK293T) cells transfected with c*CaSR*/pcDNA5/FRT were specifically labeled by the antiserum (Fig. 2A), while HEK293T cells transfected with the empty vector pcDNA5/FRT were not (Fig. 2B). We also found immunoreactivities in chicken kidney, which was reported to express CaSR^25^, by the antiserum (Fig. 2C). We then observed CaSR expression in chicken taste buds labeled by vimentin, the molecular marker for chicken taste buds^26^ (Fig. 2D). We also observed double-positive taste cells expressing both vimentin and CaSR in taste buds (Fig. 2E). We confirmed that specific immunosignals within chicken taste buds were not seen when the antiserum was omitted (Fig. 2F).

### Extracellular Ca^2+^ and Mg^2+^ activated cCaSR in a dose dependent manner

**Figure 3:**
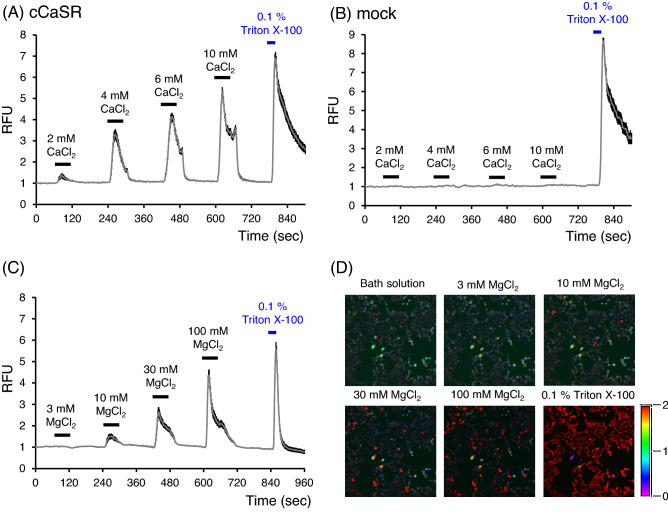
CaCl_2_ and MgCl_2_ activated cCaSR-expressing HEK293T cells in a dose dependent manner. (**A**) Representative data of the ratios of relative fluorescence units (RFUs), the index of cytosolic Ca^2+^ concentration, to the baseline value after stimulus with repetitive CaCl_2_ solutions and 0.1% Triton X-100 in cCaSR-expressing cells. (**B**) CaCl_2_ solutions did not affect the RFU values in the cells transfected with empty vector (mock), although 0.1% Triton X-100 increased RFU values. (**C**) MgCl_2_ solutions increased the RFU values in cCaSR-expressing cells. Data are the means ± SE (n = 70–100 cells). (**D**) Representative changes in the index of cytosolic Ca^2+^ concentrations after stimuli, as indicated by the Fluo 4 ratio with pseudo-color expression.

After repeated stimulation with CaCl_2_, the ratio of the relative fluorescence units (RFU), reflecting the cytosolic Ca^2+^ concentration, was increased in cCaSR-expressing HEK293T cells in a dose dependent manner (Fig. 3A). The mock cells were not affected by the same extracellular Ca^2+^ stimulation (Fig. 3B). These changes were also observed by stimulation with MgCl_2_ (Fig. 3C,D).

### Several L-amino acids enhanced the cCaSR activity

**Figure 4:**
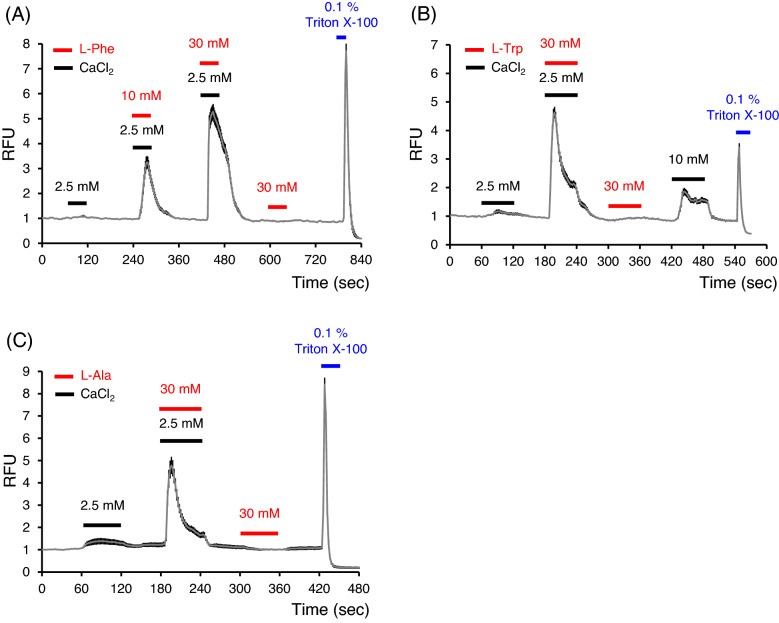
L-Phe, L-Trp, and L-Ala enhanced cCaSR activities with 2.5 mM CaCl_2_. (**A**) L-Phe enhanced cCaSR activity with 2.5 mM CaCl_2_ in a dose dependent manner. (**B**) Thirty mM L-Trp enhanced cCaSR activity with 2.5 mM CaCl_2_. (**C**) Thirty mM L-Ala also enhanced cCaSR activity with 2.5 mM CaCl_2_. RFU: relative fluorescence units, the index of cytosolic Ca^2+^ concentration. Data are means ± SE (n = 70–100 cells).

Although RFU was increased slightly by only 2.5 mM CaCl_2_, the increase was enhanced by the addition of L-Phe, L-Trp, or L-Ala (Fig. 4). However, cCaSR was not activated by these amino acids with low extracellular Ca^2+^ and Mg^2+^ bath solution (0.5 mM CaCl_2_ and MgCl_2_).

### Kokumi compounds also enhanced cCaSR activity

**Figure 5:**
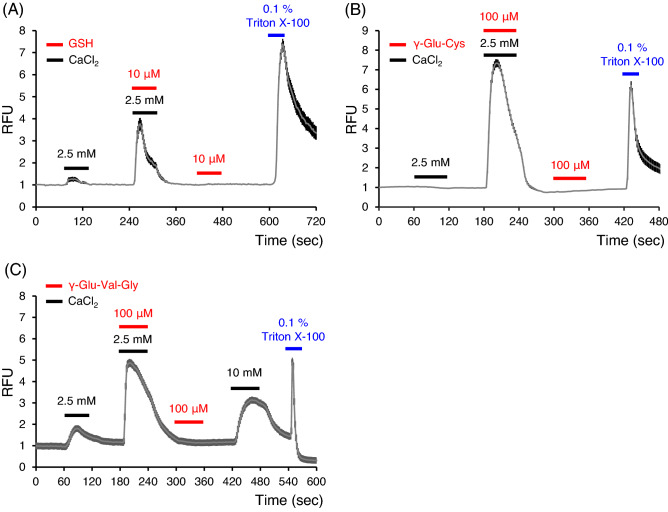
Glutathione (GSH), γ-Glu-Cys, and γ-Glu-Val-Gly enhanced cCaSR activities with 2.5 mM CaCl_2_. (**A**) Ten μM GSH enhanced cCaSR activity with 2.5 mM CaCl_2_. (**B**) One-hundred μM γ-Glu-Cys enhanced cCaSR activity with 2.5 mM CaCl_2_. (**C**) One-hundred μM γ-Glu-Val-Gly enhanced cCaSR activity with 2.5 mM CaCl_2_. RFU: relative fluorescence units, the index of cytosolic Ca^2+^ concentration. Data are means ± SE (n = 70–100 cells).

As did L-amino acids, *kokumi* substances such as GSH, γ-Glu-Cys, and γ-Glu-Val-Gly enhanced cCaSR activities with 2.5 mM CaCl_2_ (Fig. 5).

### NPS2143 negatively modulated cCaSR activity

**Figure 6:**
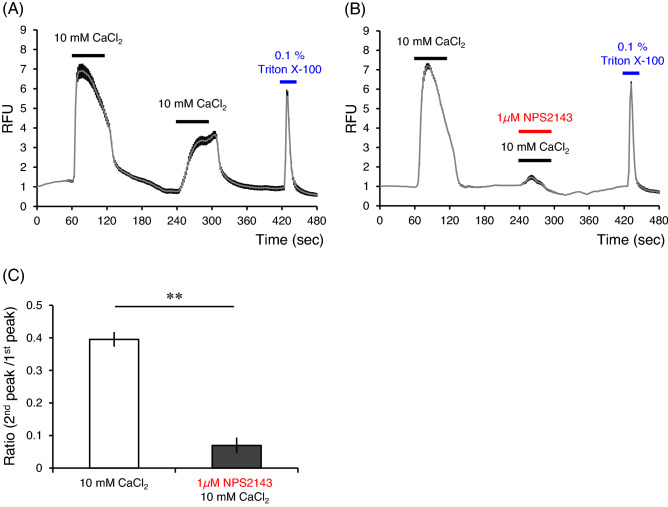
NPS2143 negatively modulated cCaSR activities with 10 mM CaCl_2_. (**A**) Ten mM CaCl_2_ activated cCaSR, but the activation by the second stimulus was desensitized. (**B**) The first stimulus by 10 mM CaCl_2_ activated cCaSR, but the activation by the second stimulus was negatively modulated by 1 μM NPS2143. (**C**) The ratios of the second peak values to the first peak values of (**A**) and (**B**). NPS2143 significantly reduced 10 mM CaCl_2_-induced cCaSR activation. RFU: relative fluorescence units, the index of cytosolic Ca^2+^ concentration. Data are means ± SE (n = 70–100 cells). ^**^P < 0.01 by unpaired *t*-test.

cCaSR was activated by 10 mM CaCl_2_. Repeated stimulation with 10 mM CaCl_2_ also activated cCaSR, but the second response was weaker than the first (Fig. 6A). That was thought to be attributable to the desensitization of cCaSR. In the case of 10 mM CaCl_2_ stimulus with 1 μM NPS2143, the response of cCaSR almost disappeared (Fig. 6B). We analyzed the ratio (second peak/first peak) between the responses for 10 mM CaCl_2_ only (Fig. 6A) and 10 mM CaCl_2_ with 1 μM NPS2143 (Fig. 6B). There was a significant difference in the ratio (second peak/first peak) between the two conditions (Fig. 6C). These results suggested that NPS2143 is a negative allosteric modulator of cCaSR. In this study, we confirmed cCaSR expression in cells by the first 10 mM CaCl_2_ stimulus. Although we also used NPS2143 with the first CaCl_2_ stimulus, we could not check the cCaSR-expressing cells by the second CaCl_2_ stimulus because the negative modulatory effect of NPS2143 on cCaSR persisted after washout (data not shown).

### A representative calcimimetic, cinacalcet HCl, activated cCaSR

**Figure 7:**
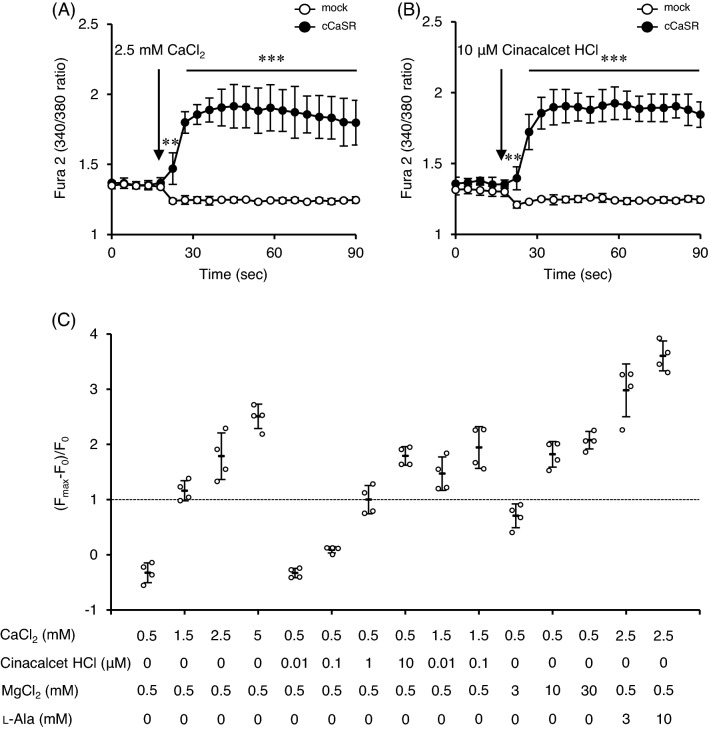
A representative calcimimetic, cinacalcet HCl, activated cCaSR. (**A**) Fura 2 fluorescence ratios in cCaSR-expressing HEK293T cells were increased by 2.5 mM CaCl_2_, but the increase was not observed in mock cells. (**B**) Ten μM cinacalcet HCl activated cCaSR. (**C**) The (F_max_−F_0_)/F_0_ after various stimuli are shown. F_0_ means the initial value of Fura 2 fluorescence ratio. F_max_ means the maximum value of Fura 2 fluorescence ratio after stimulus. These values are normalized by the values of 1 μM cinacalcet HCl. Data are means ± SD (n = 4 wells). ^**^P < 0.01 and ^***^P < 0.001 by two-way repeated measures ANOVA followed by unpaired *t*-test.

Fura 2 fluorescence ratios, reflecting the cytosolic Ca^2+^ concentration, were increased in cCaSR-expressing HEK293T cells after stimulation with 2.5 mM CaCl_2_ (Fig. 7A). The mock cells were not affected by the same stimulation (Fig. 7A). These changes were also observed by stimulation with a representative calcimimetic, cinacalcet HCl (Fig. 7B). Cinacalcet HCl activated cCaSR in a dose dependent manner as well as CaCl_2_, MgCl_2_, and CaCl_2_ + L-Ala (Fig. 7C). The responses of cCaSR by cinacalcet HCl were enhanced by 1.5 mM CaCl_2_ (Fig. 7C).

## Discussion

In this study, we confirmed that the expression of *CaSR* in the base of oral cavity was significantly larger than that in the tongue tip (Fig. 1). Since CaSR-positive cells were observed in vimentin-positive taste cells in the base of oral cavity, we supposed that CaSR functions as a taste receptor. Vimentin is a taste cell marker in chickens^27^, and because some vimentin-positive cells express T1R3, α-gustducin, synaptosomal-associated protein 25, and glutamate-aspartate transporter^28,29^, it is possible that CaSR may be co-expressed with these molecules in taste cells.

We cloned the *CaSR* gene from RIR strain chickens. As a result, although there were two mutations in the open reading frame (ORF) compared with that of *Gallus Gallus*, the translated amino acids were not changed from those of *Gallus Gallus*. Because *Gallus Gallus* is an ancestor species of chickens, it is estimated that CaSR is an essential receptor for living, and this important molecule has been preserved even after a long period of selection pressure. In addition, when the amino acid sequence of CaSR in chicken was compared with those in other animal species, the homology percentages were about 85% with humans, rats, and mice (data not shown). These results also implied that CaSR is an important receptor from an evolutionary standpoint.

Through Ca^2+^ imaging, we confirmed the responses of cCaSR-expressing cells to CaCl_2_ and MgCl_2_ solutions in a dose dependent manner (Fig. 3). These responses were similar to the responses of human CaSR (hCaSR)-expressing cells to CaCl_2_ and MgCl_2_ by using same method (data not shown). Because extracellular Ca^2+^ activated cCaSR-expressing cells in lower concentrations than did extracellular Mg^2+^, Ca^2+^ is thought to be the main orthosteric agonist in cCaSR as it is in hCaSR. Diaz et al. reported that cCaSR is activated by Ca^2+^ and Mg^2+^^30^. The present study showed an identical result. The binding sites of extracellular Ca^2+^ in hCaSR are five extracellular domain regions^31^. In cCaSR, only one amino acid (Ser-388) in Site 4 was mutated to Thr among these important sites. Since other Ca^2+^-binding sites were not changed, it is reasonable that cCaSR can sense Ca^2+^ as observed in the present study.

In addition, L-Phe, L-Trp, and L-Ala enhanced the responses of CaCl_2_ in cCaSR-expressing cells (Fig. 4). Under the 0.5 mM Ca^2+^ and 0.5 mM Mg^2+^ conditions, these L-amino acids did not activate cCaSR, suggesting that these L-amino acids are positive allosteric modulators of cCaSR. Thus, it is implied that these amino acids may be sensed by oral CaSR in chickens under the condition of around 2.5 mM extracellular Ca^2+^. Although a heterodimer of taste receptor type 1 member 1/3 (T1R1/T1R3), a chicken umami taste receptor candidate, is activated by L-Ala and L-Ser but not by L-Pro in a heterologous expressing system^32^, chicken T1R1/T1R3 activities for other L-amino acids have not been reported. Furthermore, because there are few T1R1/T1R3 co-expressing cells in chicken taste cells^28^, it is possible that CaSR plays a certain role as an amino acid receptor in chickens. On the other hand, since the functions of chicken T1R1 and T1R3 homodimers have not been elucidated, further studies of chicken oral amino acid receptors are needed.

In the present study, we examined the effects of three representative known *kokumi* substances, GSH, γ-Glu-Cys, and γ-Glu-Val-Gly, on cCaSR by using Ca^2+^ imaging. These compounds were found to enhance the responses of cCaSR-expressing cells to CaCl_2_ (Fig. 5). Under the low extracellular Ca^2+^ and Mg^2+^ (0.5 mM) conditions, these compounds did not show any response, suggesting that these compounds are positive allosteric modulators. Although the mutations of T145A/S170T in hCaSR reduced the responses to the potent γ-glutamyl peptide, *S*-methylglutathione, and L-Phe^33^, cCaSR does not have mutations in these portions. The present study revealed that cCaSR could receive *kokumi* substances as well as hCaSR.

We also examined the effects of NPS2143 on cCaSR and found that NPS2143 functions as a negative allosteric modulator also in the case of cCaSR. NPS2143 is expected to serve as a negative allosteric modulator of cCaSR in various studies. It has been thought that NPS2143 binds to a seven-transmembrane region of hCaSR and negatively modulates the responses to extracellular Ca^2+^. Especially, it has been reported that the mutations of Phe-668 (helix II), Arg-680 (helix III), Phe-684 (helix III), Glu-837 (helix VII), Phe-688 (helix III), and Ile-841 (helix VII) reduced the negative modulatory effects of NPS2143 to hCaSR^34,35^. These binding sites of NPS2143 are also the same in cCaSR, and there are only six amino acid residue differences in the seven-transmembrane regions between hCaSR and cCaSR. These six amino acid residues are not the known binding sites of NPS2143. Thus, it is reasonable that NPS2143 functions as a negative allosteric modulator of cCaSR.

A representative calcimimetic, cinacalcet HCl, activated cCaSR as well as other cCaSR ligands found in the present study. Furthermore, the responses of cCaSR by cinacalcet HCl were enhanced by 1.5 mM CaCl_2_. The results were identical to the previous study of hCaSR^36^. These results also suggested that the functions of cCaSR are similar to that of hCaSR and that CaSR is fundamental receptor from birds to humans.

Calcium and magnesium are essential nutrients for animals and play various roles in their bodies. There is a lot of evidence that various species, not just chickens, have an appetite and a taste sense for calcium^37^. However, the calcium taste receptor has not been fully elucidated. Tordoff et al. revealed that T1R3 is involved in the calcium and magnesium taste sense in mice^8^. Their study confirmed that T1R3 knockout mice prefer CaCl_2_ and MgCl_2_ solutions rather than water, whereas wild-type mice preferred water and were repelled by these solutions. Because T1R3 knockout mice lost their repellence to CaCl_2_ and MgCl_2_ solutions and showed a preference for them, it is supposed that there are other calcium and magnesium taste-related molecules besides T1R3. Tordoff et al. mentioned the hypothesis that CaSR may be involved in calcium taste by making a heterodimer with T1R3^9^, but there is no clear evidence as to whether CaSR functions as a calcium taste receptor. On the other hand, although chickens show an affinity for calcium, the molecular mechanisms underlying this preference have been almost unknown. The present study implied one molecular mechanism by which CaSR may be involved in calcium taste sense in chickens.

In summary, it was estimated that CaSR might function as a multifunctional receptor for calcium taste, several amino acids, and *kokumi* substances in chicken oral tissues. Further studies should be conducted to confirm whether the ligands of cCaSR are recognized as taste compounds in chickens by behavioral tests. It is hoped that the present results will propel further studies to fully elucidate calcium taste receptors, amino acid sensors, and *kokumi* flavor sensors in chickens. Furthermore, the fact that the ligands of cCaSR are almost the same as those of hCaSR implies that CaSR is an important and universal taste molecule among mammals and birds.

## Methods

### Chemicals

CaCl_2_·2H_2_O, MgCl_2_·6H_2_O, L-Phe, L-Trp, L-Ala, GSH, and NPS2143 hydrochloride were purchased from Sigma-Aldrich (St. Louis, MO, USA), and γ-Glu-Cys and γ-Glu-Val-Gly were purchased from Hokkaido System Science (Sapporo, Japan). For Ca^2+^ imaging by using Fluo 4-AM, these compounds except MgCl_2_·6H_2_O were dissolved in Ca^2+^-free and low-Mg^2+^ (0.5 mM) bath solution containing 140 mM NaCl, 5 mM KCl, 0.5 mM MgCl_2_, 10 mM HEPES, and 10 mM glucose at pH 7.4, adjusted with NaOH, to make stock solutions. MgCl_2_·6H_2_O was dissolved in a Mg^2+^-free and low-Ca^2+^ (0.5 mM) bath solution containing 140 mM NaCl, 5 mM KCl, 0.5 mM CaCl_2_, 10 mM HEPES, and 10 mM glucose at pH 7.4, adjusted with NaOH, to make stock solutions. These stock solutions were diluted with a low-Ca^2+^ and low-Mg^2+^ bath solution containing 140 mM NaCl, 5 mM KCl, 0.5 mM CaCl_2_, 0.5 mM MgCl_2_, 10 mM HEPES, and 10 mM glucose at pH 7.4, adjusted with NaOH just before each experiment. The low-Ca^2+^ and low-Mg^2+^ bath solution was used as a basic bath solution for Ca^2+^ imaging.

Cinacalcet HCl was purchased from FUJIFILM Wako Pure Chemical Corporation (Osaka, Japan). For Ca^2+^ imaging by using Fura 2-AM, cinacalcet HCl was dissolved in dimethyl sulfoxide (DMSO) and stored at − 20 °C. The DMSO stock solution (20 mM) was diluted with a low-Ca^2+^ (0.5 mM) and low-Mg^2+^ (0.5 mM) bath solution containing 140 mM NaCl, 5 mM KCl, 0.5 mM CaCl_2_, 0.5 mM MgCl_2_, 10 mM HEPES, and 10 mM glucose at pH 7.4, adjusted with NaOH.

### Animals

Fertilized eggs of the RIR strain were obtained from the National Livestock Breeding Centerʼs Okazaki station (Okazaki, Japan), and the hatching chicks were used for the present study. One-week-old chicks were used for RT-*q*PCR analysis and immunohistochemistry (IHC). A 20-week-old chick was used for c*CaSR* cloning from kidney. This study was carried out in accordance with the Guide for Animal Experiments issued by Kyushu University, the Law Concerning the Human Care and Control of Animals (Law No. 105; October 1, 1973), the Japanese Government Notification on the Feeding and Safekeeping of Animals (Notification No. 6; March 27, 1980), and the ARRIVE guidelines. It was approved by the committee for Laboratory Animal Care and Use at Kyushu University (approval no. A28-151-1).

### Reverse transcription qPCR

Total RNA was isolated from the palate, tongue tip, and the base of oral cavity with the use of FastGene RNA Premium Kit (NIPPON Genetics, Tokyo, Japan) according to the manufacture’s instructions, and genome DNA was removed with the use of DNase I (NIPPON Genetics). First-strand cDNA was synthesized using PrimeScript RT reagent Kit with gDNA Eraser (Takara Bio, Kusatsu, Japan) according to the manufacture’s instructions. For *q*PCR, PowerUp SYBR Green Master Mix (Thermo Fisher Scientific, Waltham, MA, USA) was used according to the manufacture’s instructions. Primers were designed with the aid of the NCBI nucleotide database and are shown in Table 1. *q*PCR reactions were done by StepOnePlus real time PCR system (Thermo Fisher Scientific). Each assay was performed in triplicate. To check the amplifications of unintended targets, we confirmed the single peaks in the melting curve analysis in all samples. After the RT-*q*PCR, to confirm whether the RT-*q*PCR products of the basal of oral cavity by using CaSR primer pair are identical with c*CaSR* sequence, these RT-*q*PCR products were electrophoresed by agarose gel, and the target single DNA bands around 120 bp were extracted from the agarose gel by using NucleoSpin Gel and PCR Clean-up (Takara Bio). The DNA sequences were confirmed by the Fasmac sequencing service (Atsugi, Japan).

**Table 1:** Primers used for the reverse transcription *q*PCR.

Target gene	Accession no	Primer forward	Primer reverse	Product size (bp)
*CaSR*	XM_416491.8	CACTTGCTGCTTCGAGTGTG	ATGCAGGATGTGTGGTTCTCA	120
*RPS17*	NM_204217.2	CTCCATTAAGCTGCAGGAGGA	GGTCCACTTCAATGATCTCCTGAT	92
*β-actin*	NM_205518.2	ACAGAGAGAAGATGACACAGATCAT	CAGATCCAGACGGAGGATGG	197
*GAPDH*	NM_204305.2	CCCATGTTTGTGATGGGTGTC	ATGGCATGGACAGTGGTCATA	158

### Tissue collection

Tissues were collected and frozen sections prepared as described previously with some modification^21^. The chicks were euthanized with an overdose of pentobarbital sodium solution, and the base of oral cavity and kidney were excised. For IHC analyses of the base of oral cavity, the tissues were embedded in Tissue-Tek O.C.T. Compound (Sakura Finetek Japan, Tokyo, Japan) and freshly frozen at − 80 °C. Frozen sections were cut at 10 µm thickness on a Leica CM1850 cryostat (Leica Instruments, Nussloch, Germany) and mounted on Matsunami adhesive silane (MAS)-coated glass slides (Matsunami Glass, Osaka, Japan).

### Antibodies

The primary antibodies used were custom-made rabbit polyclonal anti-chicken CaSR antiserum (XP_416491, residues 1022–1039, DNKEEVPNPEAEPSLPSA) (newly generated in this study; Sigma-Aldrich Japan, Tokyo, Japan) and mouse monoclonal anti-vimentin antibody (V9; Thermo Fisher Scientific) used in a previous study^26^. The secondary antibodies used were Alexa Fluor 488 goat anti-rabbit IgG (1:500), Alexa Fluor 488 donkey anti-rabbit IgG (1:500), Alexa Fluor 594 goat anti-rabbit IgG (1:500), and Alexa Fluor 594 goat anti-mouse IgG (1:500) (Thermo Fisher Scientific).

### Immunohistochemistry on frozen sections

A double-fluorescence IHC examination of frozen sections of the base of oral cavity was performed as described in a previous study^21^. The sections were fixed with acetone for 10 min at 4 °C and dried for 60 min at RT. After rehydration with phosphate-buffered saline (PBS), nonspecific staining was blocked by 3% normal goat serum (NGS, Vector Laboratories, Burlingame, CA, USA)/PBS with 0.3% Triton X-100 (PBS-X) for 30 min at RT. The sections were then incubated with the primary antibodies in 1% NGS/PBS-X overnight at 4 °C. The sections were rinsed with PBS and incubated with the secondary antibodies in 1% NGS/PBS-X for 60 min at RT. Following rinses with PBS, the sections were mounted using VECTASHIELD with DAPI (Vector Laboratories). A confocal laser scanning microscope system (A1R; Nikon Solutions, Tokyo, Japan) with NIS-Element AR3.2 was used for the observations.

Fresh kidney was fixed in 4% paraformaldehyde in PBS for 50 min and dehydrated with sucrose solutions (10%: 1 h, 20%: 1 h, 30%: overnight; at 4 °C). Kidney frozen block embedded in FSC 22 Frozen Section Media (Leica Biosystems, Nussloch, Germany) was sliced into 7 μm sections and placed onto silane-coated glass slides. After washing with PBS-X, then incubated in 1.5% normal donkey serum (NDS)/PBS-X for 1 h, then the sections were incubated with the primary antibody, anti-chicken CaSR antiserum (1:500), in 1.5% NDS/PBS-X overnight at 4 °C. The sections were rinsed with PBS-X and incubated with the secondary antibody, Alexa Fluor 488 donkey anti-rabbit IgG (1:500), in 1.5% NDS/PBS-X for 2 h at RT. Following rinses with PBS-X, the sections were mounted using VECTASHIELD with DAPI (Vector Laboratories). A confocal laser scanning microscope system (FluoView FV3000; Olympus, Tokyo, Japan) was used for the observations.

### Construction of plasmid

To construct the c*CaSR* plasmid, we isolated total RNA from kidney because CaSR expression in kidney is rich in other animals^38^, then synthesized first-strand cDNA by reverse transcription. The deduced ORF of c*CaSR* was amplified and sequenced. The PCR primers were designed based on the NCBI nucleotide databases of the predicted *Gallus Gallus* CaSR (XM_416491.7). The PCR product of the ORF was subcloned into the pcDNA5/FRT (Thermo Fisher Scientific) vector by using the In-Fusion HD Cloning Kit (Takara Bio). The entire sequence of *cCaSR* derived from RIR chicks was compared with the genomic NCBI database of the predicted *Gallus Gallus* CaSR.

### Cell culture

HEK293T cells (obtained from Dr. Koji Shibasaki, Gunma University, Maebashi, Japan) were maintained in Dulbecco’s Eagle’s medium (DMEM high glucose; FUJIFILM Wako Pure Chemical Corporation) containing 10% fetal bovine serum (FBS; GE Healthcare, Buckinghamshire, UK) and penicillin–streptomycin solution (× 100) (FUJIFILM Wako Pure Chemical Corporation) at 37 °C and 5% CO_2_.

### Immunocytochemistry on HEK293T cells

Culture, transfection, and immunocytochemistry on HEK293T cells were performed as described previously with some modifications^28^. HEK293T cells were transfected with either empty vector pcDNA5/FRT or c*CaSR*/pcDNA5/FRT by using ScreenFect A (FUJIFILM Wako Pure Chemical Corporation) on coverslips coated with poly-d-lysine (0.1 mg/mL). After transfection, the cells were incubated for 48 h at 37 °C in 5% CO_2_. The HEK293T cells were then washed with PBS and fixed with − 20 °C methanol for 10 min. After another wash with PBS, nonspecific staining was blocked by 3% NGS/PBS for 30 min at RT. The cells were then incubated with the rabbit anti-chicken CaSR antiserum (1:1000) in 1% NGS/PBS overnight at 4 °C.

The cells were then washed with PBS, incubated with Alexa Fluor 594 goat anti-rabbit IgG (1:500) in 1% NGS/PBS for 90 min at RT, and mounted with VECTASHIELD with DAPI. A fluorescence microscope (BZ-8000, Keyence, Osaka, Japan) was used for the observations.

### Measurement of cytosolic Ca^2+^ concentrations

For the Ca^2+^ imaging experiments by using Fluo 4, HEK293T cells were transfected with either empty vector pcDNA5/FRT for mock cells or c*CaSR*/pcDNA5/FRT by using ScreenFect A on coverslips coated with poly-d-lysine (0.1 mg/mL). After transfection, the cells were incubated for 48 h at 37 °C and 5% CO_2_. The cells were then loaded with 1.25 μM Fluo 4-AM solution for 30 min at 37 °C and 5% CO_2_ in the dark. Fluo 4-AM solution was prepared according to the manufacturer’s instructions (Dojindo Laboratories, Kumamoto, Japan). The coverslips were washed with the low-Ca^2+^ and low-Mg^2+^ bath solution, and Fluo 4 fluorescence was measured in that solution using a confocal laser-scanning microscope (A1R; Nikon Solutions). The coverslips were mounted in a chamber connected to a gravity flow system to deliver various stimuli. Chemical stimulation was applied by running the bath solution containing various chemical reagents. The activity of the intracellular Ca^2+^ indicator (Fluo 4) was confirmed by responses to 0.1% Triton X-100.

For the Ca^2+^ imaging experiments by using Fura 2, HEK293T cells were transfected with either empty vector pcDNA5/FRT for mock cells or c*CaSR*/pcDNA5/FRT by using ScreenFect A on 96-well clear bottom black plate (Thermo Fisher Scientific) coated with poly-d-lysine (0.1 mg/mL). After transfection, the cells were incubated for 48 h at 37 °C and 5% CO_2_. Then, we loaded Fura 2-AM solution per well in accordance with the manufacturer’s manual (Dojindo Laboratories). After incubation for 30 min at 37 °C and 5% CO_2_, calcium imaging was performed using a multimode microplate reader (FlexStation 3, Molecular Devices, San Jose, CA, USA). The assay was carried out at about 37 °C, and stimulating solutions dissolved in the low-Ca^2+^ and low-Mg^2+^ bath solution were injected automatically. Cell activity was analyzed by the value of the ratio of fluorescence intensity excited at 340 nm and 380 nm before and after injection.

### Statistical analysis

The data are expressed as means ± SD or SE. Statistical analyses were performed using the one-way factorial ANOVA followed by post-hoc Tukey HSD test, unpaired *t*-test, or two-way repeated measures ANOVA followed by unpaired *t*-test using the IBM SPSS Statistics (Version 27, Armonk, NY, USA), and differences with *P* values < 0.05 were considered significant.
